# Diffusion patterns of new anti-diabetic drugs into hospitals in Taiwan: the case of Thiazolidinediones for diabetes

**DOI:** 10.1186/1472-6963-11-21

**Published:** 2011-01-31

**Authors:** Yu-Wen Wen, Weng-Foung Huang, Yue-Chune Lee, Ken N Kuo, Chia-Rung Tsai, Yi-Wen Tsai

**Affiliations:** 1 Clinical Informatics and Medical Statistics Research Center, Chang Gung University, Kwei-Shan Tao-Yuan, Taiwan; 2 Institute of Health and Welfare Policy, National Yang-Ming University, Taipei City, Taiwan; 3 Institute of Population Health Sciences, National Health Research Institutes, Miaoli County, Taiwan; 4 National Institute of Cancer Research, National Health Research Institutes, Tainan, Taiwan

## Abstract

**Background:**

Diffusion of new drugs in the health care market affects patients' access to new treatment options and health care expenditures. We examined how a new drug class for diabetes mellitus, thiazolidinediones (TZDs), diffused in the health care market in Taiwan.

**Methods:**

Assuming that monthly hospital prescriptions of TZDs could serve as a micro-market to perform drug penetration studies, we retrieved monthly TZD prescription data for 580 hospitals in Taiwan from Taiwan's National Health Insurance Research Database for the period between March 1, 2001 and December 31, 2005. Three diffusion parameters, time to adoption, speed of penetration (monthly growth on prescriptions), and peak penetration (maximum monthly prescription) were evaluated. Cox proportional hazards model and quantile regressions were estimated for analyses on the diffusion parameters.

**Results:**

Prior hospital-level pharmaceutical prescription concentration significantly deterred the adoption of the new drug class (HR: 0.02, 95%CI = 0.01 to 0.04). Adoption of TZDs was slower in district hospitals (HR = 0.43, 95%CI = 0.24 to 0.75) than medical centers and faster in non-profit hospitals than public hospitals (HR = 1.79, 95%CI = 1.23 to 2.61). Quantile regression showed that penetration speed was associated with a hospital's prior anti-diabetic prescriptions (25%Q: 18.29; 50%Q: 25.57; 75%Q: 30.97). Higher peaks were found in hospitals that had adopted TZD early (25%Q: -40.33; 50%Q: -38.65; 75%Q: -32.29) and in hospitals in which the drugs penetrated more quickly (25%Q: 16.53; 50%Q: 24.91; 75%Q: 31.50).

**Conclusions:**

Medical centers began to prescribe TZDs earlier, and they prescribed more TZDs at a faster pace. The TZD diffusion patterns varied among hospitals depending accreditation level, ownership type, and prescription volume of Anti-diabetic drugs.

## Background

New medical technology improves quality of life and extends longevity, but it also accounts for most of the growth in health care spending [1,2] and influences the health care market by shifting demand [3]. The rate at which a new medical technology is adopted by care providers (i.e., the speed of diffusion) affects the short-term and medium-term benefits and costs. Understanding the diffusion of technology and its impact on the overall health care expenditure is essential to health care financing and insurance coverage.

Diffusion is the informal process by which innovations spread to members of a social system [4]. It is determined by the attributes of the innovation itself, communication over time, and attributes of the social system. In Rogers' (1962) diffusion theory, a continuum of cultural norms from traditional to modern is proposed to explain how a given social system responds to a new idea across space and time [5]. The social system's adoption of an innovation is determined by individuals' knowledge, attitudes, and behavior toward the innovation [5]. It may be driven by information transfers [6], normative pressures [7], and network effects [8]. Rogers subcategorized members of a social system into the innovators, early adopters, early majority, late majority, and laggards.

Incorporating Roger's diffusion theory and measuring the number of adopters into his model, Bass found that the spread of new product information resembled the spread of contagion as described in epidemiology and that was distributed through social forces to potential buyers who adopt the new product over time [9,10].

Researchers have investigated the diffusion or adoption patterns of high-cost new medical technologies such as computer topography (CT) scans [11], magnetic resonance imaging (MRI) machines [11-14], neonatal intensive care units (NICU) [15], laparoscopic instruments [16-21], and technologies for specific diseases or surgery [22-24]. Diffusion of the expensive technologies is largely influenced by their characteristics, profitability, prestige value, familiarity, and investment costs [25]. Other factors include organizational characteristics of the adopter, insurance reimbursement, public regulations, malpractice concerns, competitive or cooperative interactions among providers, and demographics [26]. However, the most important factors for acquisition of new technology and frequency of use are insurance, regulation, and provider interaction [26].

Large quantities of less expensive items routinely purchased by hospitals may add up to a greater impact on overall costs than expensive but less utilized items [27]. Prescription drugs are one such category of items. Although new drugs sometimes reduce the total cost of treatment [28], replacing older, cheaper drugs by newer, more expensive drugs accounts for a substantial portion of the rise in health care expenditures [29] and deserves further study.

Few studies have examined the diffusion of new drugs [24,30-32] and the impact on health care market. Past studies have focused on pricing and quantity of pharmaceuticals, the competition between brand name products and generics [33-36], the effect of generics on the price and market share of brand name products [37-47], as well as the effect of new drug diffusion on the quality differentiation and marketing efforts [34,48]. In contrast to macro-market analyses, little research has been conducted on new drug diffusion patterns in hospitals. Hospitals vary in size, policies, production function, market power, and input factor costs, all characteristics that can contribute to new drug diffusion. Thiazolidinediones (TZDs) are oral medications that increase insulin sensitivity and cardiovascular complications in patients with type 2 diabetes mellitus [49]. Rosiglitazone and pioglitazone are two of such agents approved by the U.S. Food and Drug Administration in 1999. Their use spread quickly throughout the U.S, where their use to treat type 2 diabetes increased from 2.2% in 1997 to 5.4% in 2001 [50]. In 2001, outpatient prescriptions of oral antidiabetic agents totaled 91.8 million [51]. In Taiwan, BNHI data indicated that the total annual prescriptions for both TZDs increased from 1.42% of all anti-diabetic agents in 2001, the year the BNHI first listed them in the national formulary for reimbursement, to 10.78% in 2003 [52].

This study analyzed nationwide prescription claims data to characterize how a new DM drug (TZD) is adopted and utilized in different hospitals how soon the hospital starts prescribing TZD, how fast the prescription grows, and to what extent the number of prescriptions increases. We also sought to investigate whether hospital characteristics can explain the variation of diffusion.

### Characteristics of health care and the pharmaceutical market in Taiwan

In Taiwan, a national health insurance (NHI) program covers the healthcare of over 99% of Taiwan's citizens. It provides comprehensive health coverage, including prescribed medications. Hospitals in Taiwan provide both inpatient and outpatient services. Under NHI, there is no gatekeeper or strict referral system, and thus patients are free to choose the health care provider. Because outpatients are usually the hospitals' main source for possible inpatients, hospitals are often motivated to compete for outpatient patients with clinics. Drugs in Taiwan are prescribed by physicians and are dispensed by hospitals or clinics. In 2007, 67.29% of NHI total medical care expenses were devoted to outpatient care, 30.14% being devoted to pharmaceuticals [53]. Hospitals, however, claimed 61.18% of the total NHI outpatient care expense and 70.21% of the outpatient pharmaceutical expenses [53]. In Taiwan, hospital behavior varies by hospital accreditation level, hospital capacity and organization. Hospitals accredited as medical centers usually have more than a thousand beds, while those accredited as regional and district hospitals have more than a hundred. Medical centers see more than four times and 27 times the number of DM patients on an outpatient basis than regional and district hospitals, respectively [54]. They have also been found to be early adopters of TZD and found to have higher penetration rates than hospitals at other accreditation levels [54].

## Methods

### Study design

The entries of rosiglitazone and pioglitazone into Taiwan were marked by their inclusion in the BNHI official list of pharmaceuticals for reimbursement on March 1, 2001 and February 2, 2002, respectively. We combined both drugs into one drug class for analyses. In this study, each hospital was treated as a small market, a microcosm of a larger market. We first classified 580 hospitals into three groups: innovators and early adopters (top 0-16%), early majority (17%-50%), late majority and laggards (51%-100%). Because 35% did not adopt the drugs, we combined the late majority and laggards into one group. We also examined how soon these hospitals started prescribing TZD after the BNHI listed these drugs. Due to the need for sufficient observations for each hospital, we identified 327 long-term adopters, hospitals that had prescribed TZDs for more than 12 months, for our analysis of the diffusion once they had adopted TZDs. We examined how fast the prescription numbers increased and when the prescription numbers reached a peak from March 1, 2001 (when they were included in the BNHI list) to December 31, 2005.

**Figure 1:**
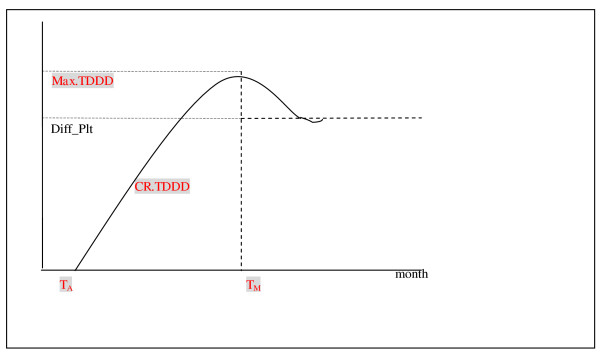
The conceptual diagram of diffusion pattern of new drug TZD in each hospital TA: time to first prescription for a TZD TM: time of peak prescription of TZD CR.TDDD: diffusion speed by month Max.TDDD: peak use of TZD Diff_Plt: plateau

To do this, we used monthly outpatient insurance claims, submitted by each hospital, to create monthly trends in the prescription of anti-diabetic drugs for each hospital. Monthly insurance claims can be used to chart diffusion, characterized by time to first prescription for a TZD (TA), diffusion speed by month (CR.TDDD), peak use (Max.TDDD), and plateau (Diff_Plt). As shown in Figure 1, the first use of a TZD in hospitals generally started later than the date the drugs were listed on the BNHI formulary.

### Sample Definition

This study focused on all hospitals (n = 651) treating patients with Type 2 diabetes (ICD9CM = 250.X2) in Taiwan between 2000 and 2005. We did not include clinics because they usually lacked economies of scale and were more likely to have contracts with a specific pharmaceutical company or limited number of pharmaceutical companies, and would, therefore, not be representative of competitive markets for pharmaceutical companies. We further excluded 71 hospitals with monthly prescriptions of anti-diabetes drugs fewer than four. These hospitals were different from the hospitals included in the study in terms of economies of scale, prescription volume and prescription pattern [54]. We were left with 580 hospitals to study time to TZD adoption. They were classified into (1) innovoators or early adopters (n = 93), (2) early majority (n = 197) and (3) late majority and laggards (n = 290). Because of considerations regarding scale, we focused our attention on 327 long-term adopters, hospitals which had prescribed TZDs for more than twelve months. The other 253 hospitals were short-term adopters or non-adopters, and they were excluded from analysis for the diffusion pattern after adoption. These hospitals were different from those long-term adopters with regard to accreditation level and ownership (Table 1). They tended to have higher pharmaceutical concentration (Herfindahl-Hirschman Index), fewer physicians on staff, and lower volumes of Defined Daily Doses (DDD) of diabetic prescriptions. They also started prescribing TZDs approximately three years after the entry of new drug, far later than those included in analysis (mean, 15 months). Defined daily doses (DDD), a WHO statistical measure of drug consumption, are used to standardize the comparative usage of various drugs between themselves or between different health care environments. A DDD is the assumed average maintenance dose per day for a drug used for its main indication in adults [55].

**Table 1:** Characteristics of adopters

	Long-term adopters		Short-term adopters		Non-adopters		P-value
				
	N	%	N	%	N	%	
Total	327	100	48	100	205	100	
Accreditation level							
Medical Center	18	5	0	0	1	0	
Regional Hospital	68	21	2	4	8	4	
District Hospital	241	74	46	96	196	96	0.0000^4^
Ownership							
Public Hospital	67	20	7	15	17	8	
Non-profit Hospital	65	20	3	6	4	2	
Private Hospital	195	60	38	79	184	90	0.0000^4^
BNHI Regions							
Taipei	78	24	12	25	26	13	
Northern	49	15	6	12	23	11	
Central	81	25	5	10	40	20	
Southern	49	15	9	19	40	20	
Kao-Ping	59	18	14	29	72	35	
Eastern	11	3	2	5	4	1	0.0003^4^

	**mean**	**Std**	**mean**	**Std**	**mean**	**Std**	
Herfindahl-Hirschman Indexes^1 ^(HHI)	0.339	0.137	0.45	0.180	0.57	0.223	0.0000^5^
Number of doctors who prescribed TZD drug	34.02	66.97	4.86	5.16	-	-	
Initial TZD use after TA (month)	15.19	11.61	36.79	16.00	-	-	
Total DDD of DM^2 ^(DDD)	37917.47	77383.05	8468	12601	2243	3958	0.0000^5^
	**mean**	**Std**					
Maximum monthly TZD penetration^3^	0.117	0.067	-	-	-	-	
Time between initial adoption and the maximum penetration (month)	26.14	13.16	-	-	-	-	
	**mean**	**Std**					
Peak use (maximum monthly prescription of TZD in DDD)	6970.01	12678.72	-	-	-	-	
The time between initial adoption to the maximum prescription of TZD (month)	27.02	13.17	-	-	-	-	
Prescription Speed (monthly growth on TZD prescription in DDD)	252.65	374.31	-	-	-	-	
							
Maximum prescription in DDD							
25% quantile		924.83	-	-	-	-	
50% quantile		2488	-	-	-	-	
75% quantile		6454.83	-	-	-	-	
Rate of penetration							
25% quantile		39.99	-	-	-	-	
50% quantile		111.22	-	-	-	-	
75% quantile		293.97	-	-	-	-	

^1^: concentration index for hospitals in February, 2001

^2^: the total DDD of DM drugs in February, 2001

^3^: Maximum TZDs DDD/all DM drugs DDD

^4^: χ^2 ^test

^5^: one-way ANOVA

### Variables

Based on Roger's diffusion theory, hospitals could classified by when they adopted the drugs: (1) innovators and early adopters (first 16% percentile), (2) early majority (17% to 50% percentile), and (3) late majority and laggards (51% to 100% percentile). Market penetration was measured by the total number of TZD prescriptions (*TDDD*) in a hospital (*i) *within a given month *(t*). Based on the conceptual diffusion model described in Diagram 1, we used three key parameters to characterize the diffusion pattern of TZD in a hospital: (1) adoption time (*T*_*A*_), defined as the number of months between March 1, 2001 (the date when rosiglitazone was added to the BNHI reimbursement list) and the first time the hospital filed a reimbursement claim for a TZD prescription; (2) peak use of TZD prescriptions (Max.TDDD), defined as the highest number of TZD prescription claims in a month; and (3) penetration speed (CR.TDDD), defined as the monthly rate of increase of TZD prescription claims since the hospital made its first claim and calculated as Max.TDDD/(*T*_*M*_*-T*_*A*_) where *T*_*M *_is the time of TZD peak use.

Independent variables included each hospital's accreditation level (AL), ownership (OWN), and the BNHI region (REG) to which it belongs. Data were collected from BNHI database of health care institutions. Taiwan's health care institutions have four accreditation levels (AL): medical center, regional hospital, district hospital, and physician's clinic. As mentioned earlier, clinics were not included in our study. Ownership (OWN) types included public, private, and non-profit. There are six BNHI regions (REG) including the Taipei, Northern, Central, Southern, Kao-Ping, and Eastern regions. Two economic factors at each hospital were considered: (1) the total number of physicians who prescribed TZDs (NDr) each month and (2) economic scale, defined as the logarithm of total DDD of all anti-diabetic drugs prescribed in February, 2001 and denoted as *log(TDDD.DM)*) in the statistical models.

Another important factor is the pharmaceutical concentration index (*HHI_DDD*) of a hospital prior to the new drug entry, reflecting the pre-existing market share structure of anti-diabetic drugs. It was hypothesized that the new drug would confront stronger entry barriers in a less competitive market when the pre-existing use of anti-diabetic drugs was heavily dominated by certain drugs. Here we used Herfindahl-Hirschman Index (HHI) to measure the concentration of anti-diabetic drugs market share structure within a hospital before TZDs were made available. HHI, a concentration index, is commonly used by competition economists and competition authorities to measure market concentration. It is used to show the extent of market control of the largest firms in the industry and to illustrate the degree to which an industry is oligopolistic. It is defined as the sum of the squares of the market shares of the 50 largest firms (or summed over all the firms if there are fewer than 50) within the industry, where the market shares are expressed as fractions. The result is proportional to the average market share, weighted by market share. As such, it can range from 0 to 1.0, moving from a huge number of very small firms to a single monopolistic producer. Increases in the Herfindahl index generally indicate a decrease in competition and an increase of market power, whereas decreases indicate the opposite.

In our study, to calculate HHI_DDD, all 140 anti-diabetic drugs on the NBHI list were first ranked by their market share. The top 56 drugs accounted for 99% of the diabetic drug market and were individually considered as separate items. The remaining 84 brands accounted for only one percent of the market share and were combined into one item for a total of 57 drug items. For each hospital *i *in month *t*, $HHI_{it}=\sum_{j}S\_DDD_{itj}^{2}$, where $S\_DDD_{itj}^{2}$ is the total DDD share of drug item j summed over all of the drug items in the hospital. In the empirical analysis, we will multiply HHI by 100 for the estimation purposes.

### Empirical models

#### Multinomial Logistic Regression for Different Adopters (n = 580)

Hospitals characterized by the likelihoods they would adopt TZDs--innovators and early adopters (i = 2), early majority (i = 3) and late majority and laggards (i = 1) --were modeled by a multinomial logistic regression with adjustments for hospital covariates:

$$\text{Pr}\text{(y=i)=}\frac{\exp{\text{ (X}}^{\text{T}}{}_{Y_{\text{i}}})}{1+\Sigma_{\text{m=2}}^{3}\exp\;({\text{X}}^{\text{T}}{}_{Y_{\text{m}}})}$$

where *Y_i_*,i = 2,3 is the vector of regression parameters, all parameters in γ_1 _are set to be zero and X is the vector of independent variables (includes *OWN*, *REG*, 100×*HHI *and log(*TDDD.DM*)).

#### Survival Analysis for the Adoption of TZD (n = 580)

A Cox proportional hazards model was used to evaluate the association between hospital characteristics and adoption of TZD:

$$\text{h}(\text{TA})={\text{h}}_{0}{\text{(TA)exp(Z}}^{\text{T}}\alpha \text{)}$$

where *h*_0_(*TA*) denotes the baseline hazard function, and where α is the vector of regression parameters and Z (includes *OWN*, *REG*, *HHI *and *AL*) is the vector of independent variables. Stratified Cox proportion hazards model was applied based on hospital accreditation level, ownership, and region. The results from stratified Cox proportion hazard model are presented in graphs.

#### Quantile Regressions for Peak Use and Penetration Rate (n = 327)

To test the association between hospital characteristics and peak use and rate of TZD penetration, we used the quantile regression model as proposed by Koenker and Bassett [56] to estimate conditional quantile functions, where the quantiles of a response variable's distribution are specified as functions of observed covariates.

For hospital *i*, its TZD peak use and penetration rate are denoted by Max.TDDDi and CR.TDDDi, respectively. The quantile regression model pertaining to the θth quantile can be expressed as the following:

$$Q_{\theta_{1}}(Max.TDDD_{i}|X_{1i})=\beta_{\theta_{1}}^{'}X_{1i}$$

and

$$Q_{\theta_{2}}(CR.TDDD_{i}|X_{2i})=\beta_{\theta_{2}}^{'}X_{2i}$$

where β_θ1 _and β_θ2 _are the vectors of coefficients, and X_1i _and X_2i _the design matrices of the independent variables for Max.TDDD_i _and CR.TDDD_i_, respectively. X_1i _includes hospital characteristics of TA, log (TDDD.DM), NDr, CR.TDDD, AL, OWN, and REG. X_2i _includes hospital characteristics of TA, log (TDDD.DM), HHI, NDr, AL, OWN, and REG. Q_θ1_(Max.TDDD_i _X_1i_); Q_θ2_(CR.TDDD_i_|X_2i_) is the conditional quantile functions of Max.TDDD_i _and CR.TDDD_i _and θ_i _(i = 1,2) is the quantile (25%, 50% and 75%). The k_th _element of vector β_θ1 _and β_θ2 _represents the marginal effect of the k_th_

$$\beta_{\theta_{1}\;k}=\frac{\partial Q_{\theta_{1}}(Max.TDDD_{i}|X_{1i})}{\partial X_{1ik}}$$

and

$$\beta_{\theta_{2}\;k}=\frac{\partial Q_{\theta_{2}}(CR.TDDD_{i}|X_{2i})}{\partial X_{2ik}}$$

where β_θ1k _is the marginal change in the θ_th _conditional quantile as a result of a change in X_1ik_. β_θ1k _, marginal effect, varies over the different quantiles. For a given set of X_ji_, we estimate a set of coefficents {β_θj_, θj = 0.25, 0.5, 075, j = 1,2}.

## Results

Table 1 shows the characteristics of the 580 hospitals: long-term adopters (n = 327), short-term adopters (n = 48) and non-adopters (n = 205). Most of the hospitals were district hospitals (n = 241, 74% long-term adopters; n = 46, 96% short-term adopters; n = 196, 96% non-adopters) and privately owned (n = 195, 60% long-term adopters; n = 38, 79% short-term adopters; n = 184, 90% non-adopters). Long-term adopters had an average of 34 physicians per hospital prescribing TZDs and initially adopted TZDs on average 15 months from the time the drugs were listed by the NBHI. Their average monthly use of anti-diabetic drugs in the month prior to TZD's entry into the market was 37917.47 DDDs. Their use of TZDs peaked on average to 0.117 (=11.7%) of all anti-diabetic drugs with mean peak time being 26 months. The mean peak prescription number (maximum DDD of TZD prescribed) was 6970 DDDs per month. The monthly growth of TZD prescription numbers (i.e., penetration speed) after the introduction was 252 DDDs/month. It took 27 months to for usage to peak in these long-term adopters. For short-term adopters and non-adopters, the average use of anti-diabetic drugs in the month immediately before TZD entry were 8468 DDDs and 2243 DDDs, respectively.

Table 2 shows that non-profit hospitals and private hospitals were more likely to be innovators and early adopters (OR = 4.12, 95%CI = 1.56 to 10.87; OR = 3.91, 95%CI = 1.64 to 9.36) and early majority (OR = 4.77, 95%CI = 1.87 to 12.20; OR = 3.38, 95%CI = 1.41 to 8.09) than public hospitals. Hospitals with higher HHI were less likely to be innovators and early adopters relative to late majority and laggards (OR = 0.95, 95%CI = 0.92 to 0.98) and early majority relative to late majority and laggards (OR = 0.97, 95%CI = 0.94 to 0.99). Hospitals with larger prior antidiabetic prescription capacity in log scale were more likely to be innovators and early adopters relative to late majority and laggards (OR = 1.93, 95%CI = 1.50 to 2.49) and early majority relative to late majority and laggards (OR = 1.64, 95%CI = 1.30 to 2.08).

**Table 2:** Multinomial logit regression on TZD adoption

	innovators and early adopters v.s. late majority and laggards			early majority v.s. late majority and laggards		
Total	OR^2^	95% CI		OR^2^	95% CI	
Ownership						
Ref: Public Hospital						
Non-profit Hospital	4.12	(1.56 10.87)	**	4.77	(1.87 12.2)	**
Private Hospital	3.91	(1.64 9.36)	**	3.38	(1.41 8.09)	*
BNHI Regions						
Ref: Taipei						
Northern	0.51	(0.19 1.39)		0.47	(0.18 1.23)	
Central	0.85	(0.35 2.06)		0.58	(0.24 1.40)	
Southern	1.31	(0.51 3.33)		0.90	(0.36 2.23)	
Kao-Ping	0.91	(0.38 2.20)		0.73	(0.32 1.68)	
Eastern	0.30	(0.03 2.93)		0.96	(0.22 4.09)	
Herfindahl-Hirschman Indexes^1 ^(unit: HHI*100)	0.95	(0.92 0.98)	**	0.97	(0.94 0.99)	*
log (total DDD of DM in February, 2001)	1.93	(1.50 2.49)	***	1.64	(1.30 2.08)	***

^1^: concentration index for hospitals in February, 2001

^2^: odds ratio

***: p < 0.0005 **: p < 0.005 *:p < 0.05

Table 3 shows that introduction of TZDs started early in medical centers (8.28 months after NBHI listing), non-profit hospitals (12.22 months), and those in the Taipei region (13.12 months). The peak use was lower in public hospitals (7.82%) and hospitals in the Northern region (8.18%). The time lag between initial TZD prescriptions and the peak use was shorter in district hospitals (24.69 months), private hospitals (25.49 months) and hospitals in the Southern region.

**Table 3:** Patterns of adoption and diffusion of TZD in 327 long-term hospitals

		Initial time (month)		Peak Penetration^1^		Time to Peak (month)		Monthly growth (DDD/month)	
					
	N	mean	std	Mean	std	mean	std	mean	std
Total number		15.19	11.61	0.1166	0.0667	26.69	13.42	252.65	374.31
									
Accreditation level									
Medical Center	18	8.28	10.31	0.1034	0.0402	34.67	12.25	1275.25	692.43
Regional Hospital	68	10.97	9.52	0.0985	0.0516	31.68	11.07	410.71	298.44
District Hospital	241	16.90	11.77	0.1227	0.0711	24.69	13.57	131.67	176.87
									
Ownership									
Public Hospital	67	15.94	10.96	0.0782	0.0469	25.63	12.54	302.22	446.00
Non-profit Hospital	65	12.22	10.18	0.1155	0.0543	31.40	12.45	467.35	513.15
Private Hospital	195	15.93	12.15	0.1302	0.0712	25.49	13.74	164.04	238.14
									
BNHI Regions									
Taipei	78	13.12	9.96	0.1204	0.0657	28.19	13.10	314.77	475.98
Northern	49	16.82	12.21	0.0818	0.0465	23.53	13.52	274.29	397.45
Central	81	16.93	12.21	0.1333	0.0683	26.95	12.85	203.92	284.37
Southern	49	14.08	11.22	0.1164	0.0689	22.90	11.79	294.41	374.88
Kao-Ping	59	15.29	12.86	0.1189	0.0718	28.83	15.56	201.43	328.36
Eastern	11	14.36	8.72	0.1104	0.0566	33.64	8.16	163.19	182.91

^1^: Peak Penetration among patients receiving non-insulin anti-diabetic agents

Based on the Cox Proportional Hazards Models, the adoption of TZDs was negatively associated with pharmaceutical concentration levels (HR = 0.02, 95% CI = 0.01 to 0.04) (Table 4). District hospitals adopted TZDs more slowly than medical centers (HR = 0.43, 95%CI = 0.24 to 0.75). Non-profit hospitals adopted TZD faster the public hospitals (HR = 1.79, 95%CI = 1.23 to 2.61). Hospitals in the Northern (HR = 0.60, 95%CI = 0.41 to 0.89) and Kao-Ping regions (HR = 0.58, 95%CI = 0.41 to 0.82) started prescribing TZDs later than those in the Taipei region. Most estimates remained the same in the analysis on TZD adopters, except in the case of private hospitals, which, after this analysis, became earlier adopters than public hospitals (HR = 2.14, 95%CI = 1.49 to 3.06). We checked the assumption of proportional hazards and they met the constant proportional hazards assumption (all p-values were > 0.05). We did not include the number of doctors who prescribed the drug and the economic scale (log(TDDD.DM) in the final model estimation because they were highly correlated with the hospital's accreditation level.

**Table 4:** Cox proportional hazards models on initial time of adoption of TZD Unit:(month)

	580 hospitals (total) initial time to adoption of TZD (month)				327 hospitals (adopters only) initial time to adoption of TZD (month)			
	HR^1^	95% CI	p-value		HR^1^	95% CI	p-value	
HHI in February, 2001 (unit: HHI*100)	0.02	(0.01 0.04)	0.0000	***	0.07	(0.03 0.18)	0.0000	***
								
Accreditation level								
ref: Medical Center								
Regional Hospital	0.75	(0.43 1.32)	0.3000		1.01	(0.56 1.81)	0.9700	
District Hospital	0.43	(0.24-0.75)	0.0024	**	0.53	(0.3 0.94)	0.0260	*
								
BNHI Regions								
Ref: Taipei								
Northern	0.6	(0.41 0.89)	0.0090	*	0.65	(0.44 0.96)	0.0280	*
Central	0.88	(0.63 1.23)	0.4500		0.82	(0.58 1.14)	0.2300	
Southern	0.75	(0.52 1.09)	0.1200		0.83	(0.57 1.21)	0.3300	
Kao-Ping	0.58	(0.41 0.82)	0.0015	**	0.98	(0.69 1.4)	0.9200	
Eastern	0.69	(0.37 1.27)	0.2300		0.7	(0.37 1.32)	0.2600	
Ownership								
ref: Public Hospital								
Non-profit Hospital	1.79	(1.23 2.61)	0.0019	**	1.87	(1.26 2.78)	0.0015	**
Private Hospital	1.35	(0.94 1.94)	0.0950		2.14	(1.49 3.06)	0.0000	***

^1^: hazard ratio; HR < 1 means the factor inhibited adoption and an HR > 1 means the factor accelerated adoption

***: p < 0.0005 **: p < 0.005 *:p < 0.05

Results from the quantile regressions showed the differential marginal effects of various factors on the penetration speed in different quantiles (Table 5). In three quantiles, the penetration speed was not significantly associated with the time lag to adoption of TZD or hospital-level pharmaceutical concentration index. It was, however, positively associated with prior anti-diabetic prescription capacity (*log(TDDD.DM)*) and the number of physicians prescribing TZD. The effect of prescription capacity on penetration speed was larger in the higher quantiles than lower quantiles (25%Q: 18.29; 50%Q: 25.57; 75%Q: 30.97). The effect of number of physicians who prescribed TZDs followed a similar pattern (25%Q: 2.65; 50%Q: 3.14; 75%Q: 4.25). Accreditation level was found to exert a significant effect on penetration speed in the 50% quantile and 75% quantile, but not in the 25% quantile. In general, regional hospitals (50%Q: -326.44) and district hospitals (50%Q: -394.48; 75%Q: -486.58) had slower penetration growth than medical centers, but not in the 25% quantile. The effects of geographic region on penetration speed were inconsistent. In general, hospitals in the eastern regions had slower pentetration speed than those in other regions, although this difference was not significant in the 75% quantile. Private hospitals had significantly higher penetration speed than public hospitals. Non-profit hospitals also had higher penetration speed than public hospitals except in the 75% quantile.

**Table 5:** Quantile regressions on monthly growth of TZD prescriptions, (n = 327 long-term adopters)

	Speed of TZD prescription (Monthly Growth on Total DDD of TZD)			Speed of TZD prescription (Monthly Growth on Total DDD of TZD)			Speed of TZD prescription (Monthly Growth on Total DDD of TZD)		
	25% quantile			50% quantile			75% quantile		
	**Coefficient**	95% CI		**Coefficient**	95% CI		**Coefficient**	95% CI	
Intercept	57.14	(-73.90 149.35)		143.87	(-125.85 474.82)		248.98	(-142.24 1045.51)	
Initial time to adoption of TZD in month	0.60	(-0.17 0.99 )		0.47	(-0.81 0.82)		0.13	(-0.57 1.86)	
log (total DDD of DM in February, 2001)	18.29	(12.14 27.75)	*	25.57	(14.74 34.87)	*	30.97	(17.17 40.80)	*
HHI in February, 2001(unit: HHI*100)	-7.88	(-55.35 45.67)		10.81	(-78.56 90.25)		67.73	(-46.51 180.98)	
Number of doctors who prescribed TZD	2.65	(0.71 4.49 )	*	3.14	(1.44 5.20)	*	4.25	(1.19 6.30)	*
Accreditation level									
Ref: Medical Center									
Regional Hospital	-193.39	(-355.21 396.19)		-326.44	(-577.59 -35.84)	*	-295.57	(-962.53 159.11)	
District Hospital	-249.47	(-410.38 451.57)		-394.48	(-647.42 -101.94)	*	-486.58	(-1351.85 -32.69)	*
NHI Regions									
Ref: Taipei									
Northern	-20.33	(-30.27 -1.70)	*	-23.94	(-58.11 52.29)		-11.05	(-93.18 51.03)	
Central	2.85	(-4.73 15.90)		18.64	(-34.77 49.89)		-40.12	(-89.09 -2.39)	*
Southern	6.93	(-10.92 20.61)		7.90	(-23.38 45.70)		-22.49	(-67.47 119.84)	
Kao-Ping	-4.05	(-18.10 10.18 )		2.64	(-37.65 32.81)		-69.48	(-112.26 -16.17)	*
Eastern	-38.15	(-60.45 -27.91)	*	-71.45	(-104.30 -50.74)	*	-114.75	(-171.58 - 36.97)	
Ownership									
Ref: Public Hospital									
Non-profit Hospital	74.52	(42.13 95.49)	*	103.08	(30.75 130.09)	*	73.89	(-0.08 145.26)	
Private Hospital	53.95	(30.05 71.65)	*	66.10	(12.52 91.35)	*	77.15	(37.58 112.26)	*

*: the 95% confident interval doesn't contain zero

Table 6 presents the factors that had significant effects on peak use across three quantile levels. Longer time lag between NBHI listing and the first TZD prescription at a hospital was associated with lower peak use at a hospital (25%Q: -40.33DDD, 50%Q: -38.65DDD, 75%Q: -32.29DDD). Peak use was also positively associated with the speed of TZD prescription increase at a hospital (25%Q: 16.53DDD, 50%Q: 24.91DDD, 75%Q: 31.50DDD). Unlike the scale effect of prescription capacity on penetration speed (Table 4), a larger volume of anti-diabetic prescriptions before TZD introduction was not associated with TZD peak use. Medical centers had greater peak use than regional and district hospitals in the 75% quantile. Non-profit and private hospitals had higher peak use than the public hospitals across three quantiles. Hospitals in the northern regions significantly lower peak use of TZDs than those in the Taipei region across three quantiles.

**Table 6:** Quantile regressions on peak monthly TZD prescriptions in DDD, (n = 327 long-term adopters)

	Peak Monthly Prescriptions in DDD				Peak Monthly Prescriptions in DDD				Peak Monthly Prescriptions in DDD			
	25% quantile				50% quantile				75% quantile			
	**Coefficient**	95% CI			**Coefficient**	95% CI			**Coefficent**	95% CI		
Intercept	-11683.46	(-25849.01	4891.88)		3087.64	(-1755.33	9708.63)		6092.86	(3040.05	13060.79)	*
												
Initial time to adoption of TZD in month	-40.33	(-90.40	-21.74)	*	-38.65	(-60.14	-18.79)	*	-32.29	(-40.34	-25.03)	*
												
log (total DDD of DM in February, 2001)	144.32	(-23.53	501.21)		63.94	(-103.01	252.24)		-7.11	(-134.58	55.07)	
												
Number of doctors who prescribed TZD	56.75	(-0.63	128.80)		41.67	(23.84	71.43)	*	35.51	(14.66	104.83)	*
												
Prescription Speed (monthly growth on TZD prescription; DDD/month)	16.53	(10.99	18.80 )	*	24.91	(20.61	28.60)	*	31.50	(28.04	35.07)	*
Accreditation level												
Ref: Medical Center												
Regional Hospital	9572.52	(-9434.71	21541.35)		-3241.95	(-8473.35	4505.39)		-5666.94	(-20463.60	-1502.91)	*
District Hospital	8507.36	(-11661.53	22343.97)		-3801.32	(-9289.80	2804.17)		-5868.08	(-13770.06	-1838.18)	*
NHI Regions												
Ref: Taipei												
Northern	-1017.83	(-1835.78	-547.86)	*	-866.80	(-1422.52	-133.63)	*	-451.32	(-625.48	-233.11)	*
Central	70.82	(-410.31	368.95)		-95.32	(-304.72	272.10)		-28.60	(-251.57	172.36)	
Southern	-487.75	(-1144.62	-11.28)	*	-495.10	(-1015.13	-54.84)	*	-451.29	(-842.53	-2.09)	*
Kao-Ping	-69.25	(-839.40	553.97)		179.82	(-143.65	823.57)		507.19	(105.50	1048.41)	*
Eastern	497.82	(-405.36	1013.11)		-63.27	(-652.49	235.63)		-375.01	(-651.40	-28.58)	*
Ownership												
Ref: Public Hospital												
Non-profit Hospital	2478.04	(1309.10	5472.91)	*	1081.43	(497.90	1751.43)	*	725.85	(321.59	987.01)	*
Private Hospital	2521.98	(1138.68	4492.53)	*	872.32	(344.70	1456.89)	*	391.32	(159.27	658.54)	*

*: the 95% confident interval doesn't contain zero

## Discussion

In this study, we analyzed the diffusion patterns of a new class of anti-diabetic drugs, TZDs, in the micro-markets of hospitals across Taiwan, including how soon hospitals started prescribing these drugs after they were listed on the national formulary, how quickly the drugs penetrated each hospital as a small health care market, and the peak use between March 1, 2001 and December 31, 2005. Our study showed the median time to adoption was about 15-20 months after formulary listing, and after 50 months, 20 to 40% of hospitals had still not adopted the drug. The adoption rates seemed very low. According to our another analyses, the market share of TZD in the second year after formulary listing was over 5% and in 2005 it was over 7%[57]. The low adoption rates could possibly be related to the characteristics of Taiwan health care market and the reimbursement rules of National Health Insurance. In Taiwan, medical centers have a very high volume of DM outpatient visits. The Bureau of National Health Insurance imposes a threshold (ceiling reimbursement) for each outpatient visit, highest for medical centers and lowest for the clinics. When the claim exceeds the threshold, they are reviewed by the Bureau and are likely to be rejected without proper justification. In addition, because DM is a chronic disease, most patients receive 30-day prescriptions of their DM drugs each time. In order to avoid risk of a claim being rejected for reimbursement, most clinics would have had less incentive to prescribe TZD because its unit price was much higher than the unit prices of the other oral antidiabetic agents. Nevertheless, most importantly, our study showed that health care provider characteristics presented a potential access barrier to new drug for Type II DM in Taiwan.

We found that the market penetration of TZDs varied substantially by individual hospital characteristics, such as a hospital's prior pharmaceutical structure, accreditation level, and ownership type. The adoption of the new drug class TZDs in a hospital was highly correlated with the hospital's prior pharmaceutical structure (measured by modified Herfindahl-Hirschman Index). Although the statistics of our modified HHI cannot be interpreted directly according to general threshold, they indicate to some extent the pattern of market structure of anti-diabetic drugs in Taiwan. Our study showed that early adoption was more likely if the physicians in a particular hospital were in the habit of prescribing multiple anti-diabetic drugs for their patients. This prescribing pattern was also associated with shorter time lag between adoption and new drug availability. The rate of growth of TZDs in a hospital was influenced by the hospital's economic scale of anti-diabetic drugs and the number of physicians prescribing TZDs. The speed of TZD penetration was not significantly associated with how quickly a hospital adopted TZDs. These findings indicate that the rate of market penetration was largely determined by prescription capacity and the herding effect. Peak use was, in contrast, influenced by how quickly hospitals adopted TZDs but not prescription capacity. In addition, faster penetration speed was linked to higher peak use.

**Figure 2:**
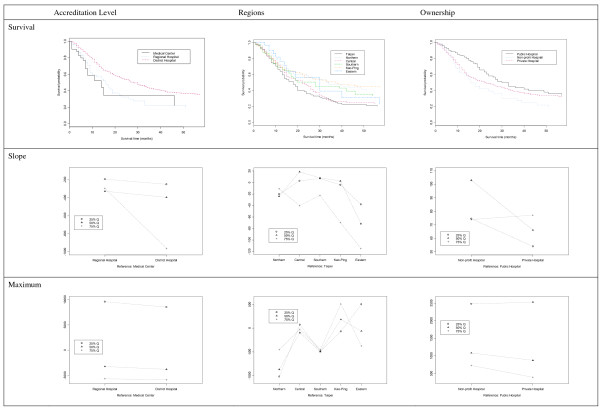
**The diffusion pattern of new DM drug among hospitals in Taiwan**.

Our results indicated that the adoption and diffusion of new TZDs was highly associated with accreditation level and hospital ownership (Figure 2). Medical centers tended to adopt the new drugs earlier than the other hospitals. In Taiwan, medical centers have a high volume of outpatient visits and low concentration HHIs. Their relatively large prescription volumes may be one reason medical centers were more likely to prescribe new drugs. Another possible explanation is that pharmaceutical industry targets medical centers the most for promotion because of the large volume of anti-diabetic prescriptions at these institutions.

Medical centers and other high-volume hospitals had a lower peak use (10.34%) than other types of hospitals. This finding demonstrates that, although a less-concentrated market is easier to enter, new drugs may still face intense internal competition within the hospital, especially if they are not a new and unique treatment for a disease. Because the BNHI regularly analyzes drug expenditure data, hospitals that spend more on medications, including medical centers that care for patients with more severe diseases, are pressured to cut costs and prescribe less expensive drugs. In addition, it is possible that new drugs had been used in the medical centers. These centers are sometimes involved in clinical studies of drugs that yet to be listed on BNHI formulary. These medical centers may, therefore, be much faster to adopt new drugs because of their prior experience.

With regard to ownership types, public hospitals were slower to adopt TZDs than non-profit and private hospitals and had lower rates of increase and peak number of prescriptions for TZDs. Public hospitals' low pharmaceutical concentration index did not facilitate the new drug entry into this market sector. This phenomenon is worthy of further investigation because public hospitals are different from non-profit and private hospitals in several ways. First, many public hospitals are subject to more restrictive policies and regulations such as collective bargaining. New drugs may not easily enter a public hospital without the agreement from other hospitals in the same alliance. The purchasing committees are composed of physicians and staff coming from different hospitals and backgrounds and may take some time to reach consensus on adopting new drugs into the collective purchase list. Second, public hospitals receive financial subsidies from the government and their staff members are government employees paid set salaries. These physicians have less financial incentive to prescribe expensive medicine, unlike physicians in private hospitals who may have gained directly or indirectly through increased prescribing of new drugs.

Most research on diffusion of technology has assumed that consumers are homogenous in their consumption behavior. This approach does not provide a comprehensive picture of market penetration. Many factors may influence how new drugs are adopted and diffused into a market, but research in these areas are lacking. Our study examined the diffusion patterns of new drugs into hospitals with various characteristics. The insights gained in this study can be used to predict market penetration in different types of hospitals and provide information regarding short-term and medium-term drug utilization and healthcare cost projections.

This study has some limitations. It lacks information on the margin between reimbursed price and pharmaceutical procurement cost, which hinders the possibility of exploring the potential association between financial incentive and diffusion of new drugs. In addition, we combined all anti-diabetic drugs and both TZDs together in analyses, which may have glossed over notable clinical differences among all the drugs. On the other hand, because TZDs are chronically administered drugs, total claims will represent a mixture of new starts and refills with refills becoming the predominant share over time. Therefore, in fact, we were modeling peak use of total prescription "consumption" not penetration of the patient market in each hospital. Future analyses could be strengthened if they differentiate between new starts versus refills and take into account the patient mix. Undoubtedly, certain omitted factors, especially health-related policy interventions or events, may have influenced prescribing trends and confounded our estimates. For instance, our estimate on the adoption and the growth of prescription could be affected when NHI implemented price-cuts on pharmaceuticals. Finally, in order to analyze the diffusion pattern of the new drugs, our study excluded those health care providers which had very small volume of DM prescription. Thus, it is would limit generalization of our conclusion to the whole health care market. Our study cannot also be generalized to the diffusion pattern of health technology.

## Conclusions

In summary, our study shows that hospitals, when considered pharmaceutical micro-markets, differs substantially in their adoption and diffusion of a new drug class, TZDs, to treat type 2 diabetes. This finding suggests that the diffusion of new drugs is associated with non-medical factors regardless of their incremental therapeutic benefits or unexpected adverse reactions. In the past few years, studies on TZD have raised concerns about fluid retention and increased risk of heart failure [58-62]. Two randomized clinical trials, the DREAM trial [63] and the PROactive study [64], have found TZD to slightly increase the risk of heart failure. A recently published meta-analysis by Nissen and Wolski [65] has additionally raised concerns that TZD may also increase the risk of myocardial infarction in diabetic patients. This led to an FDA public health advisory. Given the high profile safety discussions for this class of drugs, it would be worthwhile to study the "un-adoption" of drugs following drug warnings. Therefore, future research could examine when and how diffusion of new drugs is desired or beneficial to the public.

## Competing interests

The authors declare that they have no competing interests.

## Authors' contributions

YWW carried out the research design, analysis and draft parts of the manuscript. WFH initiated the original idea, background information, research design and draft parts of the manuscript. YCL participated in the design and drafted the parts of the manuscript. KNK participated in literature review, identified the research design issues, participated in coordination, and reviewed the manuscript. CRT participated in research design, data management and analysis, and helped organize the manuscript. YWT initiated original idea, carried out literature review, research design, and draft the main part of the manuscript. All authors read and approved the final manuscript.

## Pre-publication history

The pre-publication history for this paper can be accessed here:

http://www.biomedcentral.com/1472-6963/11/21/prepub
